# Policy Makers', NGO, and Healthcare Workers' Accounts of Migrants' and Refugees' Healthcare Access Across Europe—Human Rights and Citizenship Based Claims

**DOI:** 10.3389/fsoc.2020.00016

**Published:** 2020-03-13

**Authors:** Hannah Bradby, Adele Lebano, Sarah Hamed, Alejandro Gil-Salmerón, Estrella Durá-Ferrandis, Jorge Garcés-Ferrer, William Sherlaw, Iva Christova, Pania Karnaki, Dina Zota, Elena Riza

**Affiliations:** ^1^Department of Sociology, Uppsala University, Uppsala, Sweden; ^2^Polibienestar Research Institute, University of Valencia, Valencia, Spain; ^3^École des Hautes Etudes en Santé Publique (EHESP), Rennes, France; ^4^National Center of Infectious and Parasitic Diseases, Sofia, Bulgaria; ^5^Institute of Preventive Medicine, Environmental and Occupational Health, Marousi, Greece; ^6^Department of Hygiene, Epidemiology and Medical Statistics, School of Medicine, National and Kapodistrian University of Athens, Athens, Greece

**Keywords:** human rights, citizenship, access to healthcare, vulnerability, migrants, refugees, Europe

## Abstract

Freely available healthcare, universally accessible to the population of citizens, is a key ideal for European welfare systems. As labor migration of the twentieth century gave way to the globalized streams of the twenty-first century, new challenges to fulfilling these ideals have emerged. The principle of freedom of movement, together with large-scale forced migration have led to large scale movements of people, making new demands on European healthcare systems which had previously been largely focused on meeting sedentary local populations' needs. Drawing on interviews with service providers working for NGOs and public healthcare systems and with policy makers across 10 European countries, this paper considers how forced migrants' healthcare needs are addressed by national health systems, with factors hindering access at organizational and individual level in particular focus. The ways in which refugees' and migrants' healthcare access is prevented are considered in terms of claims based on citizenship and on the human right to health and healthcare. Where claims based on citizenship are denied and there is no means of asserting the human right to health, migrants are caught in a new form of inequality.

## Introduction

European welfare states' healthcare (Esping-Andersen, 1990; Rice, 2013) has developed on the basis of sedentism—those using the services are presumed to be a stable population that is local to where services are provided. Making healthcare universally available to citizens, regardless of who they are and of their ability to pay, through social insurance or income tax, is a powerful form of solidarity that translates basic principles of social justice (Rawls, 2001) into a manifestation of shared humanity. While definitions of accessibility and affordability of healthcare have been debated, this is not the interest of this paper. Our interest is how access to healthcare as an expression of solidarity plays out in the face of migration-driven diversity (Crepaz and Lijphart, 2008) accompanied by xenophobic populist politics (Bradby et al., 2017, 2018).

From the inception of the European Union, healthcare remained a national responsibility, with great variation in how it was achieved across Europe. European treaties have confirmed healthcare provision to be the responsibility of individual member states, despite that provision depending on the movement of people, goods and services that are subject to European Law (Legido-Quigley et al., 2008). The national basis of healthcare has been further challenged by the increasing mobility of patients from the 1990s onwards, traveling voluntarily for leisure, retirement, in pursuit of employment and education, as well as forced migrants fleeing natural disaster, conflict, oppression, poverty and lack of opportunity^1^. Migrants who retired and retirees who migrate within Europe who, as older people tend to need regular access to healthcare services, have tested the limits of European social citizenship in terms of which healthcare access rights are transferrable across national borders (Dwyer, 2001). The European Court of Justice upheld the rights of patients to obtain care in other member states, paid for by their home country in 2006 (Legido-Quigley et al., 2007), allowing citizens to assert cross-border patient mobility rights. Nationally configured, European healthcare for citizens has adapted to transnational mobility, for instance with the right to cross-border reimbursements for treatment. So while healthcare provision is still organized on the basis of sedentarist assumptions, the new normal of widespread migration (Castles and Miller, 2009) and other forms of mobility (Urry, 2007) are acknowledged at the level of European citizens' rights. However, the extent to which these rights have actually been claimed remains limited (Peeters, 2012): despite the theoretical possibility of seeking better quality care in another country, European citizens continue to prefer to access care in their local area in the main (Winblad et al., 2012).

The declaration of the universal nature of human rights includes a right to “medical care and necessary social services”^2^ with the right to health asserted to be “fundamental”^3^. The assertion of the human right to health tends to be made when citizenship claims cannot be asserted, that is to say with respect to non-European and undocumented migrants. Human rights and citizens' rights share historical roots in liberal individualism with the cosmopolitan discourse of human rights aiming to abolish the distinction between the rights of citizens and of non-citizens on which modern states were founded. Rights-based claims seek to equalize the status of citizens and non-citizens within and across states, at least along some significant dimensions (Nash, 2009, p. 1079). The human right to health has been evoked as a means of asserting access to healthcare, as well as the conditions for a healthy life, and to argue for treatment to be made available to marginalized migrants (Deblonde et al., 2015).

The ideal of human rights equalizes access and opportunities for citizens and non-citizens, and yet the language of fundamental human rights gives little purchase on structures of social and economic inequality (Nash, 2009, p. 1080). The declaration of the universal nature of human rights is an ideal that does not address the observation that having one's rights violated is far from a universal experience (Farmer, 2003, p. 1490). Any campaign for health and human rights has to also address access to social and economic resources, including healthcare. The question of how an egalitarian and anti-racist healthcare strategy might be articulated and organized (rather than just claimed) in terms of human rights is unclear. Paradoxically, far from un-doing inequality, the effect of making human rights claims may be to complicate citizenship as a rights-bearing status to create new forms of inequality (Nash, 2009).

In making the claim that human rights-based claims create new forms of inequality, Nash (2009) sets out a typology of citizenship to cover those without rights who might rely on human rights claims. Citizens are divided into “super-citizens” and “marginal citizens,” with “super-citizens” asserting citizenship rights across a number of national settings, due to material and/or culture capital. Marginal citizens' ability to assert their claims is hindered by the disadvantages of poverty and discrimination based on classism and racism, as illustrated in the UK with the so-called Windrush scandal^4^ of 2018. “Quasi citizens” are long-term residents of a state who have employment and some associated welfare rights, but not national voting rights and would include migrant workers from Turkey or Russia, resident in a European country. “Sub-citizens” are migrants who lack paid employment and access to state benefits and would include refugees whose asylum case is under consideration and the family dependents of quasi-citizens. Un-citizens are those who have no recognized status in their country of residence and no means of applying for asylum, even if they have been resident, working and paying taxes for an extended period of time as undocumented migrants (Nash, 2009).

While statutory migration regimes classify migrants differentially according to the intention and cause of their journey, with concomitant legal implications, the actual migrant may be unaware of these classifications and/or disagree with them. A migrant who has failed to lodge an asylum claim in a way that is legally recognized, may nonetheless feel herself to be a “genuine refugee” rather than an “undocumented migrant” irrespective of the legal assignation. Similarly, the distinction between a sub-citizen and an un-citizen is conceptually drawn in order to explore regimes of rights and is not necessarily an identity that people themselves would embrace.

Migrants' and refugees' access to health and to healthcare varies across the member states of Europe (Rechel et al., 2011) and across the migration and legal status of the individual (Pace, 2011). Human rights obligations toward undocumented migrants are met only partially, or not at all, in the majority of Member States. Countries that have restrictive healthcare entitlement for undocumented migrants tend to adopt “internal” migration control by restricting access to welfare benefits and public resources rather than “external” border control (Cuadra, 2012). The ability to claim a human right to health is uneven and far from fulfilled in legal and regulatory frameworks in Europe (Pace, 2011; Cuadra, 2012).

This paper draws on interview material with healthcare providers, policy makers and non-government organization (NGO) workers from 10 European countries who were asked about the health care needs of vulnerable migrants and refugees they were working with and the factors facilitating or preventing access. While working with healthcare for migrants, these interviewees they did not necessarily know the migration status of the healthcare user; they described their clients as “vulnerable” in as much as accessing healthcare was difficult. Throughout this paper the more general term “migrant” is used, to cover refugees with and without a recognized asylum claim as well as undocumented migrants.

These interviews were undertaken in people's native languages and in their workplaces in the immediate aftermath (2017–2018) of the so-called refugee (or migration) crisis. The material offers very limited background data to contextualize the interview excerpts in terms of details of the speakers' work with migrants. Furthermore, although we are referencing ideas of citizenship and human rights, we do not seek to describe the constitutional and legal differences across the European settings where interviews were undertaken.

This paper asks how healthcare providers, policy makers and NGO workers described the difficulties that refugees and migrants faced in accessing healthcare. Do healthcare providers, policy makers and NGO workers talk in terms of human-rights to health or do they talk in terms of nationally-bounded citizens' rights? Is it possible to see the types of stratification that Nash describes and are the difficulties of accessing healthcare reinforced or tackled by human rights discourse? Can we see evidence of new forms of inequality?

## Methods

Interviews were undertaken as part of the MigHealthcare project (https://www.mighealthcare.eu/) on vulnerable migrants' access to health services in Europe, which brought together scholars, service providers and policy makers across 10 European countries. A common interview guide was drafted in English, translated into all the different languages of the consortium and used by the partners to conduct the focus group discussions and individual interviews (see Box 1 for the interview guide).

Box 1 **Focus group and individual Interview guide.**

∘ What has been your involvement with health care provision for migrants/refugees?
∘ In your experience, what do migrants say that they need most in term of physical, mental and dental care?
∘ Is it possible for local services to address these needs?
∘ What sort of tools or services would help you to better assist migrants/refugees to effectively address the issues mentioned above?
∘ Do you think local communities would assist? Do you think local communities have a role in migrant integration and if they do, can you elaborate on that?
∘ Is there a need to guide migrants on how to use the health care system?

Before each interview information sheets in the appropriate local language were distributed, outlining the study, describing the interview process and the subsequent processing of the interview material. Interviewees signed a consent form giving permission for the pseudonymous use of their words. As soon as interviews were undertaken and recorded, they were pseudonymized, so that individuals cannot be neither identified in nor associated with interview excerpts.

## Data Collection

Between November 2017 and April 2018, 21 focus group discussions and 22 individual interviews were conducted with health care providers, policy makers and representatives from NGOs, including volunteer workers, in the 10 countries of the consortium—Austria, Bulgaria, Cyprus, France, Germany, Greece, Italy, Malta, Spain, and Sweden—see Table 1. Where it proved impossible to convene people for focus group discussions due to incompatible timetables and interviewees' competing commitments, then individual interviews were undertaken. The table below provides a summary of the number of focus group discussions and interviews and the type of participants in each country.

**Table 1:** Interviews per country.

**Country**	**Focus group**	**Individual interviews**	**Participants**
Malta	1	8	NGOs; Policy Makers; Health care professionals
Austria	2	6	NGOs; Policy Makers; Health care professionals
Italy	3	–	NGOs; Policy Makers; Health care professionals
Spain	3	–	NGOs; Policy Makers; Health care professionals
Greece	3	–	NGOs; Policy Makers; Health care professionals
Germany	2	2	NGOs; Policy Makers; Health care professionals; Social Workers
France	3	–	NGOs; Policy Makers; Health care professionals
Cyprus	2	2	NGOs; Policy Makers; Health care professionals
Sweden	–	4	NGOs; Policy Makers; Health care professionals
Bulgaria	2	–	NGOs; Policy Makers; Health care professionals

## Data Analysis

Full transcripts of interviews that were conducted in English were shared. Where interviews were conducted in a language other than English, a detailed summary was produced by following a template that was shared across the consortium. The use of summaries in English enabled researchers to access data produced in languages other than English. The disadvantage of this approach, as opposed to using than full translated transcripts, is that some of the nuance of meaning in the original source language is lost, but this is weighed against the advantage of being able to include views from 10 different languages and national settings. While fully translated transcripts would have captured more detail and nuance, time and budget constraints required a quicker means of sharing material across languages. This means of translating and summarizing material allowed for a thematic analysis, but did not support a narrative or content analysis. Since the analysis was undertaken from summaries (as well as transcripts), the specific terms used in the original discussions and interviews could not always be checked. In particular the lack of specificity around the terminology of types of migrants (refugees, asylum seekers, rejected asylum seekers, forced migrants, undocumented migrants) could not be confirmed. In the text below the term “migrant” is used as to cover the range of different types of migrants and refugees.

The interview and focus group material was coded and organized in terms of content. The first order coding was conducted by Adele Lebano and Sarah Hamed and shared with the consortium for validation and used as the basis for a research report^5^. This analysis is looking for healthcare access being considered as a national resource (accessible through citizen-like claims) and as a matter of human rights, which would not rely on citizenship.

## Results

### Healthcare as a National Resource With Access Determined by Migration Status

Our interviews suggest that for some migrants, healthcare was regarded as a national resource, tightly aligned to migration governance. Undocumented migrants were said to actively avoid contact with healthcare services. This was more than being unable to claim access to healthcare, in that some migrants were reported to make strenuous attempts to avoid healthcare providers due to a fear of being reported to the migration authorities. According to a hospital health manager in France:

They don't want to come to the hospital. They are afraid of being registered, of being picked up by the police.

Disclosing the migration status of a patient to the authorities would go against the professional and personal ethics and ethos of many healthcare professionals; nonetheless some migrants' fears made service provision almost impossible. A migrant's fear that healthcare provision was closely allied to migration governance led to difficulties for a hospital based doctor in Austria: a patient would not communicate for fear of being reported, despite being extremely ill. According to this Austrian doctor:

A black man came to the hospital at 3 am with fever of 40 degrees. He refused to name his country of origin as someone has told him that if he would do so, he would be refused the examination. This was also the reason why he would not say a word during the examination which made the whole procedure very difficult.

Far from asserting a human right to health, migrants were avoiding accessing healthcare for fear of triggering investigations of their legal status. This finding is not surprising given the welfarist^6^ and populist politics that denies the entitlement of non-citizens to access services. Nonetheless, it is striking that migrants were said to be endangering their own health to avoid healthcare contact.

In some cases, migrants' avoidance of healthcare was attributed to the Dublin regulation^7^ which states that an asylum claim must be lodged in the first European country of contact. Healthcare workers in both Greece and Malta said that migrants were seeking to pass through and claim asylum in another location such as Germany, the UK or Sweden and so did not want to risk being waylaid. Migrants apparently feared jeopardizing a subsequent asylum claim by becoming visible to the national authorities in a country that they were trying to pass through. The avoidance of healthcare suggests that accessing healthcare is seen as making a claim on a national resource, linked with migration status and its governance, rather than being a human right based on clinical need.

### Healthcare as a Human Right

Some of the healthcare workers that we interviewed had been actively engaged with asserting healthcare access as a right that was not linked with citizenship. This was emphasized by the following healthcare provider, working in a NGO in Greece who asserted that, at least officially, migrants, regardless of their legal status, could access services, saying:

We should also stress that in terms of legal access we have laws that have liberated many, many things. Today we generally have a better access to health care and everyone has access to the health care system without problems, regardless of their status…This right has been granted to migrants/refugees, something that wasn't happening until now. And we fought for it!

Campaigning to secure legal rights to access healthcare regardless of migration status is a commitment to healthcare access as a human right. But the existence of a formal right of access does not, of course, imply that people can actually access services that meet their needs appropriately. Since the provision of healthcare in Europe remains a national, regional and (in some cases) municipal responsibility, the existence of a legal right at international or national level may do little to secure access locally. Indeed the same NGO healthcare provider quoted above who pointed out that access rights existed legally regardless of migrants' status, described how workers in Greek government agencies and Citizens' Service Centers were not “informed enough to respond to the Law's requirements.” Similarly, in Austria, Greece, France, Italy and Germany interviewees suggested that even with formal access, migrants and refugees did not get good access to healthcare. In Malta, a community development officer told us that “some service providers are not sure what the entitlements are when family members present different legal statuses” while in Italy healthcare providers were said to be in need of training in legal aspects of care provision for refugees. One healthcare professional told us that:

A very key point is the need of training the health service staff, at different levels-from the people in charge of the front office and administration issues to the linguistic mediators up to the specialist practitioners. There is the need to increase their knowledge and skills on the law regulating the migrants' health rights, on epidemiology, the main infectious diseases, medical anthropology, ethno-psychiatrics etc.

The promotion of access to healthcare as a fundamental human right creates new forms of inequality in the sense that a human rights claim has to be fulfilled by national or regional agencies who have national or local interests to protect. Given the high profile of anti-migrant “welfarist” politics across Europe, migrants' assumption that contact with healthcare agencies will lead to registration with migration authorities is understandable; moreover evidence of the reluctance of some healthcare professionals to provide healthcare to migrants of uncertain legal status, confirms such assumptions.

### Uneven Access Across Countries

Our interviewees were engaged with ways of providing healthcare to migrants and some of them were explicitly committed to health as a human right, such that a lack of citizenship or of residency papers should not debar access. Healthcare workers based in countries that receive a lot of migrants—Greece and Italy—described different experiences compared with those receiving large numbers of asylum applications—Germany and Sweden. Interviewees in Greece emphasized the humanitarian nature of their work, and stressed that healthcare provision was constructed in order to respond to the acute needs of migrants who wanted to transit to another country.

In France, which is a country both of transition and of destination for migrants, healthcare workers noted the dual difficulties for migrants claiming health as a human right and getting access to healthcare through citizenship claims.

While NGOs were able to provide services and promote access in specific settings, national structures and agencies could promote or prevent access to healthcare services on a wider scale. For migrants arriving in Cyprus by boat, initial health assessments and information about the health system were made available, whereas those arriving over land through the northern, Turkish part of the island got neither the assessment nor the information. The arrivals via the sea were treated as refugees, with access to services as a human right, whereas arrivals by foot were not treated as refugees, but had no citizens' rights either.

### Migration Regulation Preventing Healthcare Access

International attempts to regulate migration flows were described as exacerbating the problems of providing adequate healthcare for migrants. A German psychiatrist with long experience of working with migrants said that the new arrivals in 2015 exhibited worrying new disorders such as post-traumatic stress and anxiety regarding their unclear legal status and uncertain life circumstances. The agreement between the European Union and Turkey to restrict migrants in closed camps outside Europe was said to have a damaging effect on the health of new arrivals in Europe who had managed to leave the camps, especially on their mental health.

The policy in Greece of restricting new arrivals by boats to camps on the islands where they land was criticized by staff working for local NGOs. In contrast to an earlier time when migrants with health problems could be treated in mainland hospitals, the policy of containment ignored urgent healthcare needs which could not be met in the camps.

Although during the summer there was some flexibility for those having medical issues to be transferred to the mainland, this flexibility is now gone and people must remain on islands, despite the fact that they don't have access to services.

(NGO staff, Greece)

More than preventing access to services, the restrictions imposed on migrants' movements themselves created damage to health. A NGO staff member in Greece described how treatment of migrants created health problems:

The vulnerability of these people is multiplied due to living conditions. Their hopes have disappeared. They are pent up, and cannot move on. This is the so-called trauma these people bear anyways coming from a war zone; and this trauma grows bigger during the journey, and when they reach Greece, namely Europe, after the new agreement with Turkey is magnified.

Policies that dispersed and relocated migrants were said to be damaging to migrants' health.

What we see in the refugee camps, are the people—I do not want to say the word ‘traumatized' now, but let's just say ‘[mentally] unstable' and they are pushed from one place to another. (Social worker, Germany)

According to NGO workers in Greece and Italy, the reception system of refugee camps was damaging, not only in restricting access to appropriate services but in creating conditions for mental ill health to flourish. A NGO healthcare worker and policy maker working in Greece put the negative consequences of the system of migrant reception in the following, strong terms.

I would say that if people with the best possible resilience and in the best mental shape lived under the conditions which people on Greek islands live under today, they would lose their mind in just 4 or 5 days. The second issue we have to deal with is not just the conditions in the camps, but also the migrants'/refugees' expectations that are disappointed: the stress, the uncertainty for the next day that no one knows what is going to happen tomorrow, and all these result to unwanted and disturbing behaviors.

Newly arrived migrants in this Greek's account are un-citizens, in being contained away from public healthcare facilities on island-based camps and also being denied a human rights-based claim to healthcare. This same informant described how the only way to prioritize healthcare for vulnerable migrants on the Greek mainland was sometimes to emphasize their mental ill health and, since the living conditions in the refugee camps were so poor, such a claim was in fact justifiable. She said:

To leave the islands you must appear to be vulnerable, a way to do so is by proving that you have a mental health issue. It is much harder to prove that you are pregnant when you are not, but you can claim that you suffer from mental health problems as an excuse without, in fact, lying, given that you suffer from severe discomfort due to the living conditions in the refugee camps. This is somehow the only way for someone to achieve the prioritization of the refugee's request for the examination of his/her asylum application, his/her transfer to the mainland etc.

Far from being a human right, only those with acute problems including pregnancy and severe mental distress could hope to access health services.

### Healthcare Organization Preventing Healthcare Access

Where migrants were able and willing to seek healthcare through mainstream public healthcare facilities, there are other impediments in operation. Even if healthcare staff were willing to provide services, aspects of the health system were so difficult to navigate that achieving access was extremely problematic. As a physician volunteering in a NGO in France said:

the care pathway is quite complex and … for people who do not know how to find their way around the system … in France in terms of administrative things we are quite expert in making things complicated.

In Italy a policy maker described how utterly disempowered migrants who had been “expropriated of their capacity” were and how no help was offered to support them in navigating the system. He said that the migrant “has no idea how to orient himself within a universalistic system” and furthermore

There is no giving information on the tools that a person can have to access treatment, there is only giving of treatment. Also, as regards access to relocation procedures, for example, a medical certificate is needed, but no guidance is made on how to obtain certain documents.

Unfamiliarity with a complex system may at times be further exacerbated by other intangible obstacles associated with unhelpful insensitive administrative staff and procedures (Larchanché, 2012). In Italy, a lack of coordination between the various agencies involved in health and social service provision and across different regional health systems, was also felt to prevent access. In France a waiting period of 3 months was routine before irregular foreign nationals could apply for state medical assistance (André and Azzedine, 2016), while the routine wait was said to be even longer in Malta. Where access to healthcare was dependent on an insurance-based scheme, as in Germany, migrants were effectively debarred for a period of time prior to being able to access care. Dental care is often supplied through health insurance, which was said by a hospital based doctor in France to make it “not possible” for migrants get appropriate care, given the complexities of obtaining that insurance (although alternative schemes do also exist). Since migrants were said to be preoccupied with other matters, such as achieving subsistence (mentioned in France) or coping with substance abuse (mentioned in Austria), arranging access to healthcare was not necessarily a priority. A healthcare worker in Austria said that among “young men and adolescents … alcohol and drug abuse” was a “problem to be handled with the utmost sensitivity” because the substance abuse was largely a response to “refugees” traumatic experiences as well as the stress and uncertainty they feel as asylum seekers, not having an occupation and a daily routine'. Healthcare provision was not set up to deal with the causes underlying young migrants' substance abuse.

### Lack of Linguistic and Cultural Interpretation

Very widely cited as a barrier to providing healthcare for migrants and refugees was the lack of interpreters and of cultural mediators, mentioned by workers in Austria, Greece, Italy, Malta, Spain, Germany and Cyprus. As an Italian policy maker put it “the biggest problem to access healthcare services are the linguistic barriers.”

The lack of linguistic interpretation services prevented verbal communication since, as a Greek healthcare provider said, “there isn't a single government official who can speak English, Arabic or any language needed.” Another healthcare provider in Greece underlined the essential nature of translation by saying “I cannot even imagine how a nurse can cope without the presence of an interpreter … to provide his/her services to an Afghan refugee.” Even if interpreters were available, they might not be trained, be skilled or experienced in healthcare work. A German psychiatrist described the difficulty of providing care through a translator who had no experience of talking about severe trauma. In Cyprus a healthcare provider described the lack of suitable translators leading forcing her to resort to poor practice out of necessity:

Because we do not have the capacity to call professionals [translators], we often use persons from within the community, or, it has happened to me on two occasions to have to communicate through the children, something that I avoid because I believe it is a big psychological burden for the child to discuss with me her mother's problems.

While the need for cultural mediators was less widely acknowledged than translators, they were said to be particularly necessary in mental health services, with a nurse working with newly arrived migrants in Sweden saying that “… for the assessment of mental health condition the cultural barrier is unbearable.” A healthcare provider noted that in Greece not all cultural mediators were appropriately educated and that “it doesn't suffice for someone to simply know the language in order to accompany a refugee to the hospital.” This provider went on to say that the need for appropriate education applied not only to cultural mediators but also to skilled healthcare professionals:

However, the most essential, of course, is doctors' education. They should be able to understand that it is entirely different to communicate and to diagnose a patient with the assistance of a third party i.e. the mediator.

The same idea was echoed by health providers in Austria and in Malta, where healthcare professionals were said to often be lacking in training and/or experience of how to work with translators and cultural mediators. A healthcare provider in Austria told us that healthcare professionals are so unaware of how to behave in translated consultations that they sometimes talked only with the interpreter, ignoring the patient. A healthcare provider in Italy said that despite significant linguistic and comprehension barriers for migrants “cultural language mediators are not considered a necessary person in helping access to public health services.”

A senior hospital nurse in Sweden described how her ward developed relationships with particular interpreters that they trusted and yet she felt that even with known translators, the amount of information that she, as an experienced clinician, could get across to a refugee with little education was highly limited.

Interpretation at healthcare encounters is mandated at national level in most European settings (Samkange-Zeeb, under review)^8^ and yet provision is poor or non-existent and largely goes un-measured as a dimension of healthcare quality. The fact that linguistic support for entry-level communication in healthcare is not routinely provided is indicative of the citizen-focussed of nature national health services. Despite good quality interpretation having been acknowledged as crucial for meaningful healthcare for decades (Flores, 2005; Bauer and Alegría, 2010), its widespread absence and poor quality suggests that anyone not versed in the local language is not seen as a legitimate or as a core healthcare user.

### Migrant as a Category of Un-patient

Healthcare providers in France and Austria described resentment toward migrants having increased over recent years. They described a sense that migrants should be grateful for whatever healthcare they receive, even if that service is restricted compared to the general population's access. The marginal position of migrants within the public healthcare system was described by a doctor volunteering with a NGO in France. She said that previously, in referral letters, patients would be designated by their diagnosis or illness or by their nationality. More recently a status as “migrant” had come to be the key designation: she went on to say that the designation of a patient as a migrant was an indication of a reluctance to treat them.

The migrant schizophrenic arrived (at the hospital), in former times schizophrenia is what mattered, but now one considers him to be a migrant, rather than being a schizophrenic. We now end up questioning the fact that he's schizophrenic because they say he's a migrant. This is quite a recent thing, in fact. […] I opened a letter this morning from a young person who had been taken care of in A&E, and had also been seen by the shrink. It is, at the beginning of the report ‘Young migrant' […] Before we used to read in hospital reports, I do not know, ‘Cameroonian' or…But now it's ‘young migrant'. This is the new category: ‘Migrants'. It is as if to say they do not really want to take care of them.

(Voluntary medical doctor in NGO, France).

### Rationed Access to Healthcare

The mass movement of people into southern Europe during 2015 and 2016, widely designated as the migrant or refugee crisis, made visible existing deficits in national healthcare systems, delivered at regional and local level and exacerbated these short-comings. In Bulgaria the lack of funding and availability of healthcare was emphasized, with the requirement to pay for treatment, disadvantaging migrants. Healthcare workers described how austerity measures that had already weakened the capacity of the public healthcare system made responding to the needs of newly arrived migrants even more difficult. Budget cuts leading to reduced staffing and equipment were noted in France, Germany, Italy, Spain, Cyprus, and Greece, as having detrimental effects at different levels of and moments in the healthcare system, as described by a NGO worker in Greece.

The economic crisis has weakened the current health care system, which was further weakened due to the refugee crisis and the incapacity of the health care authorities to respond specifically to this population. All these situations acted increasingly and led to what is seen today as deficit resulting from the combination of these situations. Many times it is more essential that we don't have the money to support the public health care system; other times that we don't have to know how; other times that we are in crisis; other times that the staff is insufficient due to cuts; other times that the staff suffers from burn-out. Each factor has a different effect each time.

Even in Germany, which was generally less affected by austerity, specific problems were noted—according to the following Medical Chamber representative, examinations that were legally mandated could not be carried out.

And there was a huge shortage, especially with regard to the X-ray examinations, because the hospital was completely overwhelmed to carry out the relevant examinations, which - according to the law - were planned.

In countries that were more affected by austerity, the reduced capacity of the healthcare system was an added difficulty for providing healthcare to migrants.

## Summary

This study of migrants' access to healthcare services in Europe, according to policy-makers, NGO workers and healthcare providers took place in the wake of the mass movement of people into southern Europe during 2015 and 2016. Healthcare workers across Europe, said that migrants were denied access to healthcare in a number of different ways, suggesting that neither citizenship nor human rights claims were effective means of claiming access. European public healthcare is organized on the assumption that patients will be able to speak the local language and will share cultural assumptions with healthcare providers. Despite the widely acknowledged necessity of linguistic interpretation and cultural mediation for meaningful healthcare encounters, such services were unavailable, unreliable or of dubious quality. The absence of reliable, good quality translation services was noted across all the countries included in our study. Despite efforts by healthcare workers to make access a human right, migrants themselves sometimes evaded contact with healthcare, apparently due to a fear of detection by national migration authorities. Such avoidance of healthcare providers is longstanding with regard to undocumented migrants (Médecins Sans Frontières, 2005) due to a (sometimes justifiable) equation of healthcare with migration regulation. When migrants did contact healthcare providers, their access was sometimes denied by individual staff due to uncertainty or reluctance to grant access and sometimes by organizational complexity impeding navigation of a healthcare pathway (Larchanché, 2012). According to our informants, individual denial of access was likely to affect migrants with uncertain legal status in particular and the absence of citizenship-based rights did not lead automatically to the granting of healthcare access as a human right. Organizational complexity of the healthcare system is highly off-putting to new arrivals and those in transit who do not share the local language or culture.

A key question is whether the difficulties that migrants have in accessing healthcare is a form of deliberate exclusion of outsiders, or an unintended side-effect of sedentarist assumptions left over from the time when healthcare systems were first established. At a time when populist anti-migrant politics are actively querying new comers' access to welfare and healthcare resources, across European countries, this question has no simple answer. However, we should note that the doctor in France quoted above suggested that referring to a patient as a migrant had come to indicate a reluctance to offer treatment. The NGO workers in the Greek islands also noted the containment of migrants away from healthcare services on the mainland, which could only be challenged in cases of acute unmet need.

## Conclusion

By attending to semi-structured interviews with healthcare workers across Europe, who may themselves have been committed to the ideal of health as a human right, we can see how little purchase it offers people who are not European citizens to claim healthcare access. The NGOs that some of our informants worked with were actively providing healthcare to migrants, which is evidence that the principle of healthcare access is accepted as a human right. But the extent to which the human right could be asserted was limited, as shown by the routine and widespread absence of cultural and linguistic translation services at healthcare encounters. The organization of healthcare as a national responsibility, with some concessions made to European mobility in recent years, makes it very difficult for migrants from outwith Europe to claim access to health services. Our evidence confirms Nash's suggestion that un-citizens and sub-citizens in Europe are caught in a double jeopardy, able to assert neither human rights-based nor citizenship-based claims to access healthcare.

Our results tend to confirm the creation of these sub-categories of citizenship occupying a no-man's land where healthcare access cannot be claimed, suggesting a need to re-conceptualize how migrants' health is understood in global and in national public health. Theorizing migrant health as a global health issue (Wild and Dawson, 2018) and a global public health good (Widdows and Marway, 2015) opens up possibilities for mediating between the logics of national political and cosmopolitan ethical discourses (Gottlieb and Mocha, 2018). As Nash points out, we should be investigating how the development of human rights within states influences the relationship between citizens and non-citizens in practice: the ability to enjoy rights is never only a matter of legal entitlement, but also depends on “social structures through which power, material resources and meanings are created and circulated” (Nash, 2009, p. 1069). This paper seeks not so much to endorse Nash's categorization, as to contribute toward developing alternative approaches to conceptualizing migrant healthcare provision, at a time when the debate tends to pit sedentarism (in the guise of nationalism or nativism) against universal human rights. Since neither citizenship-like rights nor human rights facilitate vulnerable migrants' access to healthcare, an alternative conceptualization that does not rest on statutory structures is needed to support healthcare access claims. The failure of European healthcare systems that claim to be universally available to support the most vulnerable is most obvious in the lack of availability of translation and mediation—long acknowledged as a necessary for healthcare provision.

## Data Availability Statement

The datasets generated for this study are available on request to the corresponding author.

## Ethics Statement

The studies involving human participants were reviewed and approved by Regional Ethics Committee, Uppsala (Dnr 2017/464). The participants provided their written informed consent to participate in this study.

## Author Contributions

HB first drafted the paper and took the lead in its revision in the light of other authors' comments. AL and SH commented on the first draft and vouched for the appropriate representation of the study methods. WS, AG-S, ED-F, JG-F, IC, PK, DZ, and ER read and commented on drafts and can vouch for the research in their individual member countries.

### Conflict of Interest

The authors declare that the research was conducted in the absence of any commercial or financial relationships that could be construed as a potential conflict of interest.

## Footnotes

^1^Migration governance seeks to draw a distinction between labor migrants and refugees seeking asylum, with the definition of these categories politically motivated (Zetter, 2007) and not necessarily obvious to migrants themselves.

^2^https://www.un.org/en/universal-declaration-human-rights/

^3^https://www.ohchr.org/Documents/Publications/Factsheet31.pdf

^4^https://www.theguardian.com/uk-news/windrush-scandal

^5^https://www.mighealthcare.eu/e-library

^6^Efforts to restrict access to services for new arrivals and those seen as failing to contribute sufficiently to tax funded welfare states or social insurance schemes.

^7^https://ec.europa.eu/home-affairs/what-we-do/policies/asylum/examination-of-applicants_en

^8^Samkange-Zeeb, F., Bradby, H., Samerski, S., Humphris, R. (under review). “It's the first barrier” - lack of a common language still a major obstacle when accessing/providing healthcare services across Europe. *Front. Sociol*.
